# Benefits and risks of manual hyperinflation in intubated and mechanically ventilated intensive care unit patients: a systematic review

**DOI:** 10.1186/cc11457

**Published:** 2012-08-03

**Authors:** Frederique Paulus, Jan M Binnekade, Margreeth B Vroom, Marcus J Schultz

**Affiliations:** 1 Department of Intensive Care Medicine, Academic Medical Center, Meibergdreef 9, 1105 AZ, Amsterdam, The Netherlands; 2 Laboratory for Experimental Intensive Care and Anesthesiology (L·E·I·C·A), Academic Medical Center, Meibergdreef 9, 1105 AZ, Amsterdam, The Netherlands

## Abstract

**Introduction:**

Manual hyperinflation (MH), a frequently applied maneuver in critically ill intubated and mechanically ventilated patients, is suggested to mimic a cough so that airway secretions are mobilized toward the larger airways, where they can easily be removed. As such, MH could prevent plugging of the airways.

**Methods:**

We performed a search in the databases of Medline, Embase, and the Cochrane Library from January 1990 to April 2012. We systematically reviewed the literature on evidence for postulated benefits and risks of MH in critically ill intubated and mechanically ventilated patients.

**Results:**

The search identified 50 articles, of which 19 were considered relevant. We included 13 interventional studies and six observational studies. The number of studies evaluating physiological effects of MH is limited. Trials differed too much to permit meta-analysis. It is uncertain whether MH was applied similarly in the retrieved studies. Finally, most studies are underpowered to show clinical benefit of MH. Use of MH is associated with short-term improvements in lung compliance, oxygenation, and secretion clearance, without changes in outcomes. MH has been reported to be associated with short-term and probably clinically insignificant side effects, including decreases in cardiac output, alterations of heart rates, and increased central venous pressures.

**Conclusions:**

Studies have failed to show that MH benefits critically ill intubated and mechanically ventilated patients. MH is infrequently associated with short-term side effects.

## Introduction

Manual hyperinflation (MH), also known as "bagging" or "bag-squeezing" is a frequently used maneuver in critically ill intubated and mechanically ventilated patients [1,2]. With MH, patients are disconnected from the mechanical ventilator, after which their lungs are temporarily ventilated with a manual ventilation bag. By applying a larger-than-normal volume at a low inspiratory flow followed by an inspiratory pause and expiration with a high expiratory flow, MH is suggested to mimic a normal cough. Propagation of airway secretions from the smaller toward the larger airways then allows for easy removal of airway secretions with airway suction. As such, MH could prevent airway plugging [3,4], and even promote alveolar recruitment [5].

It is far from certain whether MH truly benefits critically ill intubated and mechanically ventilated patients. In addition, disconnection of a critically ill patient from the ventilator could be seen as a rather unsafe intervention [6]. Because MH may cause short-term hyperinflation, one could even consider MH to be dangerous in hemodynamically unstable patients [7,8]. Also, MH could be disadvantageous in patients with respiratory failure. The airway pressures at the end of the MH maneuver are usually much lower than the applied level of positive end-expiratory pressure, which, in combination with airway suctioning, may promote atelectasis.

This systematic review aims to collect the evidence for the suggested benefits and risks of MH in critically ill intubated and mechanically ventilated patients. The main research questions were as follows. Does MH benefit critically ill intubated and mechanically ventilated patients with respect to pulmonary compliance, arterial oxygenation, and sputum clearance? Does MH have an effect on the duration of mechanical ventilation, length of stay in the intensive care unit, and incidence of pneumonia? What are reported side effects of MH?

### The rationale behind manual hyperinflation

Retained airway secretions may occlude the airways of intubated and mechanically ventilated patients, and, as such, cause atelectasis. This may impair oxygenation by increased intrapulmonary shunting and increase pulmonary vascular resistance. Large atelectasis may even promote development of lung injury [9]. The consequence of large atelectasis is a smaller lung available for ventilation, leading to the concept of "baby lung" ventilation [10]. Persistent presence of sputum in the airways may provide an ideal environment for colonizing organisms, finally resulting in pneumonia [11].

Frequent removal of sputum from the airways via tracheal suctioning is mandatory in critically ill intubated and mechanically ventilated patients. Under normal conditions, mucociliary transport clears the smaller airways of airway secretions. Secretions that are transported from the smaller airways into the bronchi and trachea then are removed by coughing. Critically ill patients, however, are frequently sedated and nursed in a supine position, potentially reducing mucociliary transport and promoting retention of airway secretions [12,13]. In addition, the cough reflex can be minimal or even absent in sedated critically ill patients, or they may lack force to cough efficiently. Furthermore, sputum may not be easily transported from the trachea into the translaryngeal tube or trachea cannula, and thus could remain in the larger airways. Unfortunately, with airway suctioning, only the trachea is cleared of secretions, as suction catheters cannot reach sputum in the bronchi and smaller airways. MH, as originally described in the late 1960s, was designed to enhance clearance of airway secretions [14].

### Description of the MH technique

To enhance the clearance of airway secretions, MH was supposed to include the application of a larger than normal volume (up to one and one half the size of tidal volumes delivered by the ventilator) at a low inspiratory flow (achieved by a slow compression of the ventilation bag), an inspiratory pause (to allow complete distribution of the inflated air among all the ventilated parts of the lung), and a high expiratory flow. In particular, this last element seems important and can be achieved by a complete and rapid release of the ventilation bag [15,16]. As such, MH could resemble a forceful cough, with which a forced and rapid exhalation follows a deep and slow inhalation.

It is suggested that the effectiveness of MH is dependent, at least in part, on the ratio between flows with inspiration and expiration [3,4]. With higher expiratory flows (that is, higher than inspiratory flows), sputum could propagate from distal to more proximal areas (that is, from the smaller airways toward the larger airways), where it can be easily removed through endotracheal suctioning [17,18]. It is also suggested that the use of an inspiratory pause maintains the pressure gradient for an appropriate length of time required to overcome the opening pressure of the alveoli [19].

## Materials and methods

### Search methods for identification of manuscripts about MH

Two methods were used to identify relevant manuscripts in the medical literature. First, we performed a search in the databases of Medline, Embase, the Cochrane Library, the Cochrane Database of Systematic Reviews, and the Database of Abstracts on Reviews and Effectiveness (DARE) from January 1990 to April 2012 (Additional file 1). Second, reference lists of identified and selected manuscripts were reviewed to identify additional articles.

The following key words (MeSH and text words) were used: "critical care," "intensive care," "manual hyperinflation," "hyperinflation," "bagging," and "bag squeezing." In addition, we used the key words "hyperoxygenation," "physiotherapy," and "physical therapy." We excluded studies of mechanical hyperinflation by machines, like cough-assist devices. The initial search strategy was designed for maximal retrieval, with no limitation on the type of study design to be identified. We used no restriction on language.

### Study selection

Two authors (FP and JB) independently reviewed the retrieved articles and abstracts, assessed the eligibility of each study, and resolved disagreement by consensus. Articles were selected if they reported original data from a clinical trial or an observational study. We restricted the selection of articles to those that reported on adult critically ill intubated and mechanically ventilated patients. The same authors made the final selection; we restricted the selection to articles that reported on relevant study end points, including pulmonary compliance, arterial oxygenation, sputum clearance, duration of mechanical ventilation, length of stay in the intensive care unit, and incidence of pneumonia, and only if the main objective concerned the evaluation of the MH procedure.

### Data-collection process

We extracted data from the included studies by using a data-extraction sheet. We extracted the following data: characteristics of the studies (design, setting), participants, intervention characteristics (MH technique), comparison intervention, and results of all relevant outcomes.

### Assessment of methodologic quality of individual studies

Two reviewers (FP and JB) assessed the risk of bias of the interventional studies and used the categories randomization, random sequence generation allocation concealment, description of withdrawals and dropouts, the method of and use of intention-to-treat analysis, and standardization of important co-interventions.

### Synthesis of results

The decision to combine studies in a quantitative analysis was made by assessing clinical heterogeneity (examining types of participants, interventions, and outcomes in each study).

## Results

### Study selection

**Figure 1:**
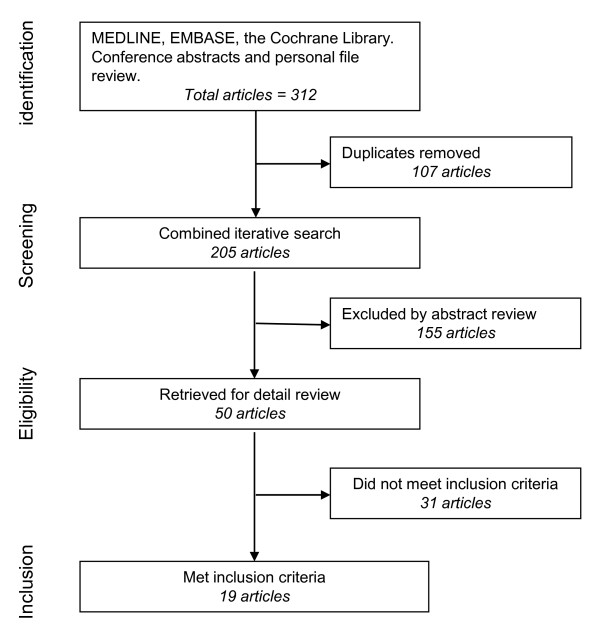
**Number of articles identified at each stage of the review process for potential inclusion in the systematic review**.

The search (Figure 1) identified 50 articles, of which 19 were considered relevant (Tables 1, 2, and 3).

**Table 1:** Studies comparing manual hyperinflation (MH) with standard care

Author [ref]	Year	Subjects	*N*	Study design	Intervention	Reported aspects of the MH maneuver	Main results
Hodgson *et al*. [26]	2000	Unselected intensive care unit patients	18	Randomized crossover trial	MH + endotracheal suction vs. standard care	Volume: - Inspiration speed: - Pause: + Expiration speed: ±	MH increased pulmonary compliance but did not affect P/F; MH increased clearance of airway secretions

Patman *et al*. [22]	2000	Postcardiac surgery patients	94	Randomized controlled trial	MH + endotracheal suction vs. standard care	Volume: - Inspiration speed: - Pause: + Expiration speed: -	MH increased pulmonary compliance and P/F

Barker *et al*. [20]	2002	Patients with acute lung injury	17	Randomized controlled trial	MH + endotracheal suction + position changes of patient vs. position changes only	Volume: + Inspiration speed: - Pause : + Expiration speed: -	MH affected neither pulmonary compliance nor P/F

Choi *et al*. [25]	2005	Patients with pneumonia	15	Randomized crossover trial	MH + endotracheal suction vs. standard care	Volume: ± Inspiration speed: - Pause: + Expiration speed: +	MH improved pulmonary compliance

Maa *et al*. [5]	2006	Patients with atelectasis	23	Randomized controlled trial	MH + endotracheal suction vs. standard care	Volume: - Inspiration speed: + Pause: + Expiration speed: ±	MH affected neither P/F nor clearance of airway secretions

Blattner *et al*. [21]	2008	Postcardiac surgery patients	55	Randomized controlled trial	MH + endotracheal suction vs. standard care	Volume: - Inspiration speed: - Pause: ± Expiration speed: -	MH improved pulmonary compliance and PaO_2 _and reduced duration of MV

Patman *et al*. [23]	2009	Brain-injury patients	144	Randomized controlled trial	MH + endotracheal suction and side lying vs. standard care	Volume: ± Inspiration speed: ± Pause: + Expiration speed: ±	MH affected neither duration of MV and length of stay in intensive care unit, nor the incidence of pneumonia

Paulus *et al*. [24]	2011	Postcardiac surgery patients	100	Randomized controlled trial	MH + endotracheal suction vs. standard care	Volume: + Inspiration speed: + Pause: + Expiration speed: +	MH did not affect pulse-oximeter oxygen saturation

Reported aspects of the MH maneuver: volume, whether larger than normal breaths were used; inspiration speed, whether a low inspiratory flow was used; pause, whether pauses were used; expiration speed, whether rapid expiratory flows were used (+, mentioned; -, not mentioned; ±, uncertain)

**Table 2:** Studies comparing manual hyperinflation (MH) with other strategies

Author [ref]	Year	Subjects	*N*	Study design	Intervention	Reported aspects of the MH maneuver	Main results
Berney *et al*. [29]	2002	Unselected intensive care unit patients	20	Randomized crossover trial	MH *vs*. hyperinflation by the mechanical ventilator	Volume: ± Inspiration speed: + Pause: + Expiration speed: ±	Both techniques improved pulmonary compliance equally; no affects on clearance of airway secretions

*Paratz et al*. [33]	2002	Patients with acute lung injury	16	Prospective observational study	MH	Volume: + Inspiration speed: + Pause: + Expiration speed: +	MH increased pulmonary compliance; P/F increased in patients with an extrapulmonary cause of acute lung injury, whereas it decreased in patients with a pulmonary cause of acute lung injury

Savian *et al*. [30]	2006	Unselected intensive care unit patients	14	Randomized crossover trial	MH vs. hyperinflation by the mechanical ventilator	Volume: - Inspiration speed: + Pause: + Expiration speed: +	MH affected neither pulmonary compliance and PaO_2_, nor clearance of airway secretions

Hodgson *et al*. [31]	2007	Unselected intensive care unit patients	20	Randomized crossover trial	MH with two different devices for hyperinflation	Volume: ± Inspiration speed: - Pause: + Expiration speed: ±	MH affect neither pulmonary compliance nor P/F; clearance of airway secretions differed between two devices

Ahmed *et al*. [28]	2010	Postcardiac surgery patients	30	Randomized controlled trial	MH vs. hyperinflation by the mechanical ventilator	Volume: ± Inspiration speed: + Pause: + Expiration speed: +	Both techniques improved P/F equally; both techniques did not affect pulmonary compliance

Dennis *et al*. [27]	2012	Patients with atelectasis	46	Randomized cross-over trial	MH vs. hyperinflation by the mechanical ventilator	Volume: ± Inspiration speed: - Pause: + Expiration speed: -	Clearance of airway secretions did not differ between the two techniques; both techniques did not affect pulmonary compliance; P/F increased after VH but it decreased after MH

Reported aspects of the MH maneuver: volume, whether larger than normal breaths were used; inspiration speed, whether a low inspiratory flow was used; pause, whether pauses were used; expiration speed, whether rapid expiratory flows were used (+, mentioned; -, not mentioned; ±, uncertain).

**Table 3:** Side effects of manual hyperinflation (MH)

Author [ref]	Year	Subjects	*N*	Study design	Intervention	Reported aspects of the MH maneuver	Main results
Singer *et al*. [8]	1994	Unselected intensive care unit patients	18	Prospective observational study	Measurements of hemodynamic parameters before and after MH	Volume: + Inspiration speed:- Pause: - Expiration speed: -	MH decreased cardiac output; MH did not affect heart rate or systemic blood pressure

Jellema *et al*. [32]	2000	Patients with septic shock	13	Prospective observational study	Measurements of hemodynamic parameters before and after MH	Volume: ± Inspiration speed: - Pause: + Expiration speed: +	MH did not affect cardiac output, heart rate, systemic blood pressure, or central venous pressure

Paratz *et al*. [33]	2002	Patients with ALI	16	Prospective observational study	Measurements of hemodynamic and respiratory parameters before and after MH	Volume: + Inspiration speed: + Pause: + Expiration speed: +	MH did not affect cardiac output, heart rate, systemic blood pressure, or central venous pressure

Paratz *et al*. [7]	2006	Patients in shock	7	Prospective observational study	Measurements of hemodynamic parameters and plasma catecholamines before and after MH	Volume: + Inspiration speed: + Pause: + Expiration speed: +	MH decreased cardiac output, and increased systemic vascular resistance, and diastolic blood pressure

Hodgson *et al*. [26]	2000	Unselected intensive care unit patients	18	Randomized crossover trial	MH + endotracheal suction vs. standard care	Volume: - Inspiration speed: - Pause: + Expiration speed: ±	MH did not affect heart rate or systemic blood pressure

Barker *et al*. [20]	2002	Patients with ALI	18	Randomized controlled trial	MH + endotracheal suction vs. position changes of patient	Volume: + Inspiration speed: - Pause : + Expiration speed: -	MH increased heart rate and systemic blood pressure; MH did not affect pulmonary artery wedge pressure

Paulus *et al*. [34]	2010	Unselected intensive care unit patients	74	Prospective observational study	Measurements of hemodynamic parameters before and after MH	Volume: + Inspiration speed: + Pause: + Expiration speed: +	MH did not affect systemic blood pressure or peripheral oxygen saturation; MH increased heart rate and end-tidal CO_2 _levels

Patman *et al*. [35]	1998	Postcardiac surgery patients	30	Prospective observational study	Measurements of hemodynamic parameters before and after MH	Volume: - Inspiration speed: - Pause: ± Expiration speed: -	MH increased central venous pressures and decreased heart rate; MH did not affect cardiac output

Reported aspects of the MH maneuver: volume, whether larger than normal breaths were used; inspiration speed, whether a low inspiratory flow was used; pause, whether pauses were used; expiration speed, whether rapid expiratory flow was used (+, mentioned; -, not mentioned; ±, uncertain).

### Study characteristics

We included 13 interventional studies (six randomized controlled trials of MH [5,20-24], two randomized crossover trials comparing MH with endotracheal suctioning [25,26], four randomized crossover trials comparing MH with hyperinflation by the mechanical ventilator [27-30], and one randomized crossover trial comparing two different manual-ventilation bags [31]), and six observational studies [7,8,32-35]. Physiological end points were respiratory mechanics [20-22,25,27-31], arterial oxygenation [5,20-24,26-28,30,31], and clearance of airway secretions [5,26,27,29-31]. Clinical end points included duration of mechanical ventilation [21,23], length of stay [21,23], and incidence of pneumonia [23]. Reported side effects included effects on heart rate and systemic blood pressure [7,8,20,26,32-34], cardiac output [7,8,32,33,35], and pulmonary artery pressures and central venous pressures [32,33,35].

### Risk of bias within studies

The risk of bias among included studies is summarized in Table 4. Quality assessment revealed that five studies did not describe concealment of allocation. Only three studies clearly reported standardization of important co-interventions to prevent performance bias. Most studies did not report the use of intention-to-treat analysis. Description of the losses to follow-up was not included in three studies. All studies were open label, because blinding of the ICU clinicians was not feasible for these types of studies.

**Table 4:** Summary of risk of bias assessment of the interventional studies

Author [ref]	Random sequence generation	Allocation concealment	Standardization of co-interventions	Intention-to-treat analysis	Description of losses to follow-up
Hodgson *et al*. [26]	+	+	±	-	+
Patman *et al*. [22]	+	+	±	-	+
Barker *et al*. [20]	+	+	±	-	+
Choi *et al*. [25]	+	-	+	-	-
Maa *et al*. [5]	±	±	±	-	+
Blattner *et al*. [21]	+	+	+	+	+
Patman *et al*. [23]	+	-	-	+	+
Paulus *et al*. [24]	+	+	-	+	+
Berney *et al*. [29]	+	+	±	-	+
Savian *et al*. [30]	+	-	±	-	-
Hodgson *et al*. [31]	+	±	±	+	+
Ahmed *et al*. [28]	+	-	±	-	-
Dennis *et al*. [27]	+	+	+	-	+

+, mentioned; -, not mentioned; ±, uncertain.

### Synthesis of results

Studies varied widely in terms of patient populations, with dissimilar reasons for (acute and/or persistent) intubation and mechanical ventilation, MH intervention, and outcome measurements. Because of the substantial clinical heterogeneity, we focused on describing individual study results, rather than using a meta-analysis.

### Physiological end points

**Figure 2:**
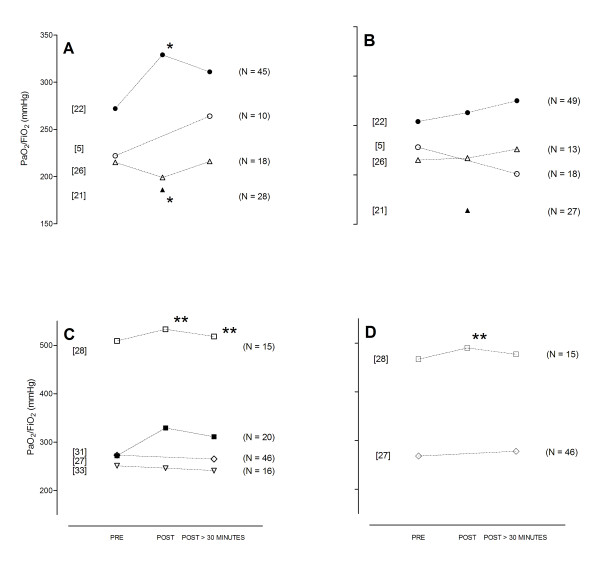
**Change in PaO_2_/FiO_2_**. These studies compared manual hyperinflation **(A) **with standard care **(B)**, or manual hyperinflation **(C) **with other strategies **(D)**, in patients after cardiac surgery [21,22,28] (preprocedure data were not reported in [21]), unselected intensive care unit patients [26,31], patients with atelectasis [5,27], and patients with acute lung injury [33]. *P < 0.05; **P < 0.001.

**Figure 3:**
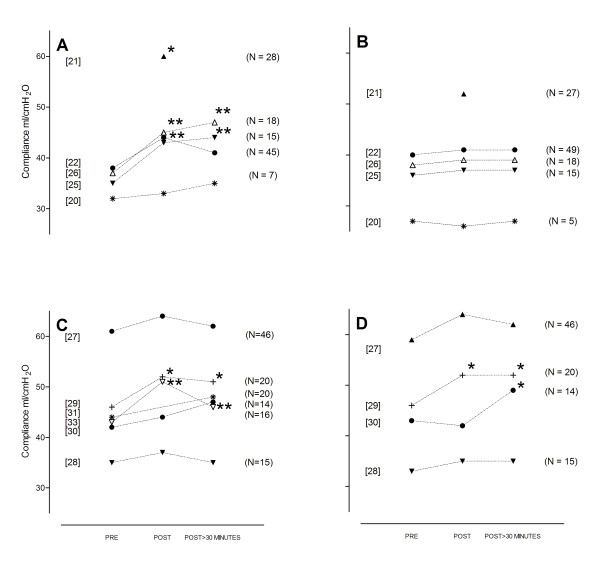
**Change in pulmonary compliance**. The studies compared manual hyperinflation **(A) **with standard care **(B)**, or manual hyperinflation **(C) **with other strategies **(D)**, in patients after cardiac surgery [21,22,28] (preprocedure data were not reported in [21]), unselected intensive care unit patients [26,29-31], patients with pneumonia [25], patients with atelectasis [27], and patients with acute lung injury [20,33]. *P < 0.05; **P < 0.001.

The results of the physiological end points for the individual studies are summarized in Figures 2 and 3. We separated studies with an active control group (for example, hyperinflation by the ventilator) from studies comparing MH with standard care.

MH improved pulmonary compliance in postcardiac surgery patients [21,22], in patients with pneumonia [25], as well as in patients with large atelectasis [5]. Notably, MH did not improve pulmonary compliance in patients with acute lung injury [20]. With respect to changes in pulmonary compliance, MH was not superior to hyperinflation by the mechanical ventilator [27-30].

MH improved arterial oxygenation in studies of postcardiac surgery patients [21,22]. However, in another study of postcardiac surgery patients, this could not be confirmed [24], nor in studies of patients with atelectasis [5], nor in a study of brain-injury patients [23]. Notably, although no effect of MH on arterial oxygenation was found in a study of patients with acute lung injury [20], another study found MH to improve arterial oxygenation in patients with indirect acute lung injury but not in patients with direct acute lung injury [7,33]. MH was not superior to hyperinflation by the mechanical ventilator with respect to arterial oxygenation [27-30].

Although studies of unselected ICU patients found MH to improve sputum clearance [26,31], this was not found in a study of patients with atelectasis [5]. Similar volumes of pulmonary secretions were mobilized with MH compared with hyperinflation by the mechanical ventilator [27,29].

### Clinical end points

MH was found to shorten the duration of mechanical ventilation in a study of postcardiac surgery patients [21]. This, however, was not confirmed in a study of brain-injury patients [23]. MH did not reduce length of stay in the ICU or hospital, in either postcardiac surgery patients [21] or brain-injury patients [23]. In addition, MH did not reduce the incidence of pneumonia in brain-injury patients [23].

### Side effects

The results of the side effects are summarized in Table 3. The majority of studies found MH not to affect systemic blood pressure and heart rate [7,8,26,32,33]. One study of patients with acute lung injury [20] and one observational study of unselected ICU patients [34] showed MH to increase heart rate and blood pressure, whereas another study in unselected patients showed MH to decrease heart rate [35]. MH was associated with a short-lived 10% to 15% decrease in cardiac output in patients with septic shock as well as in unselected ICU patients [7,8]. Conversely, this was not found in observational studies of patients with septic shock, patients with acute lung injury, and patients after cardiac surgery [32,33,35]. MH was associated with increased central venous pressures in one observational study of patients after cardiac surgery [35]. Side effects were frequently considered clinically irrelevant [7,8,20,34].

## Discussion

MH is suggested to mimic a cough so that airway secretions are mobilized from the smaller airways toward the larger airways, where they can easily be removed. As such, MH could benefit critically ill intubated and mechanically ventilated patients. We reviewed studies of diverse intensive care unit populations investigating the potential beneficial effects and side effects of MH. Most investigations consistently showed MH to be feasible and safe. MH improved pulmonary compliance, arterial oxygenation, and clearance of airway secretions, albeit not in all investigations. MH inconsistently affected clinical outcome. Side effects of MH seemed relatively infrequent.

Apart from the possibility that MH may indeed not benefit critically ill intubated and mechanically ventilated patients, studies simply may have been underpowered to detect any beneficial effect of MH, such as duration of mechanical ventilation, length of stay in the intensive care unit, and prevention of ventilator-associated pneumonia.

Studies in this review were heterogeneous in regard to patient populations, MH intervention, and outcome measurements and could not be combined in a meta-analysis. Only a few studies compared MH with standard care. In some studies, MH was compared with another strategy (for example, hyperinflation by the ventilator). Two of the retrieved studies reported the use of position changes in conjunction with MH. Multiple other strategies could have been used in conjunction with MH in other studies, such as postural drainage, vibrations, and manually assisted cough. This, however, was not clearly reported. In addition, most studies included in this review had methodologic flaws, which may have resulted in bias.

Better evidence to support the use of MH is required. Therefore, appropriately powered, well-designed, randomized controlled trials evaluating the effect of MH should be conducted. The focus of these studies should be on clinical end points, including, but not restricted to, duration of mechanical ventilation or ventilator-free days, length of stay in the ICU, and incidence of ventilator-associated pneumonia.

It is difficult to give clear recommendations on how to perform MH maneuvers. Most reports did not adequately describe how MH actually was performed. Monitoring airway pressures is feasible, but may not have priority. Notably, use of larger than normal breaths is associated with higher inspiratory airway pressures, up to 40 ± 8 cm H_2_O [36,37]. Also, almost without exception expiratory airway pressures are low, as low as 2.1 [1.4 to 3.1] cm H_2_O [37]. The most important aspect for MH to be effective may be the high expiratory flow, which can be achieved by rapid and complete release of the ventilation bag [16]. Outside the importance of conducting future studies that comprehensively describe the MH technique used, additional studies could establish how the different components of the technique are of influence on the therapeutic aims of MH.

Unfortunately, MH is frequently referred to as a maneuver to recruit lung tissue. We would like to emphasize that MH was originally designed to mimic a forceful cough. The most important principle for MH to be effective may be the high expiratory flow. Consequently, airway pressures at the end of each MH cycle are low, which may very well promote derecruitment. With the performance of MH, the necessity exists to disconnect the patient from the mechanical ventilator. Breaking the ventilatory circuit may lead to airway contamination and eventually to ventilator-associated pneumonia. Breaking the ventilatory circuit may also lead to significant airway pressure decreases and promote lung derecruitment. Finally, the delivery of larger than normal tidal volumes with MH, even for a very short time, may cause overinflation. Although scientific evidence for the existence of these potential side effects is lacking, they may very well limit adoption of MH. Derecruitment and overinflation could be harmful, especially in patients with acute lung injury, or its more severe form, acute respiratory distress syndrome. It is imaginable that derecruitment and overinflation affect the lungs differently among diverse populations of ICU patients. We suggest that MH may better recruit lung tissue in patients with easy-to-recruit lungs (for example, patients after cardiac surgery or patients with indirect lung injury) than in patients with less compliant lungs (for example, patients with direct lung injury). The dissimilar findings of investigations reviewed here are in line with this suggestion

Future studies should include patients with different pulmonary conditions and should address overinflation and derecruitment with MH. It may be necessary to add recruitment maneuvers to MH, but as far as we know, this has not been the subject of clinical studies.

The retrieved studies reported side effects of MH relatively infrequently, and most of the reported side effects were minor. Because most if not all studies were not specifically designed to detect side effects of MH, absence of reported side effects may not mean that the procedure is necessarily safe.

In a study in which patients showed a small decrease in cardiac output, it was hypothesized that this was caused by a decrease in the cardiac preload [8]. A decrease in cardiac output could therefore depend on the expertise and level of training of ICU nurses and/or respiratory therapists: a (too) large increase of the intrathoracic volume due to a (too) large tidal volume with MH may impede venous return [8,32]. It has been suggested that MH, when performed under controlled conditions and/or performed by experienced and trained ICU nurses, respiratory therapists, and/or intensivists, has negligible effects on cardiac output [32-34].

Given the paucity of data, one might simply recommend abandoning MH as a relic of good intentions with little scientific evidence, but with potential harm. However, absence of evidence does not necessarily mean evidence of absence. A pathophysiological rationale exists for MH as a secretion-clearance technique that should be tested in clinical trials. From this review of studies ,we conclude that until now, no adequately powered studies tested the hypothesis that MH benefits intubated and mechanically ventilated patients. The same also applies for the potential adverse events of MH.

Limitations exist in the way we conducted our review. First, two of the authors are ICU clinicians and frequent users of the MH procedure and may be potentially biased. Second, electronic and hand searches do not completely reflect the extent of research outcomes; for example, studies presented at congresses are more likely to contain negative reports than are studies reported in the literature. Furthermore, many studies not published in English may not be included in the most commonly used searches.

## Conclusions

MH is associated with short-term beneficial effects on lung compliance, oxygenation, and airway clearance in intubated and mechanically ventilated patients. MH is inconsistently associated with clinical benefit. MH only infrequently has been associated with side effects. It should be noted, though, that the majority of published studies were not designed to detect potential adverse events like derecruitment. Appropriately powered and methodologically sound studies of MH are needed before recommendations can be made for routine use of MH.

## Key messages

• Manual hyperinflation is a frequently used maneuver that intends to mimic a forceful cough in critically ill intubated and mechanically ventilated patients.

• Manual hyperinflation may improve pulmonary compliance, arterial oxygenation, and clearance of airway secretions.

• As such, manual hyperinflation may benefit intubated and mechanically ventilated critically ill patients.

• Side effects of manual hyperinflation seem relatively infrequent; however, most studies did not seem to be designed to detect potential adverse effects like derecruitment.

• Better evidence to support use of manual hyperinflation is required.

## Abbreviations

ICU: intensive care unit; MH: manual hyperinflation; MV: mechanical ventilation; P/F: ratio of partial pressure of arterial oxygen to fraction of inspired oxygen.

## Competing interests

The authors declare that they have no competing interests.

## Authors' contributions

FP and JMB performed the literature search and selected the relevant articles for inclusion independently. FP and JMB reviewed the selected articles. FP and JMB wrote the initial draft of the manuscript. MJS contributed to the interpretation of the results and drafting of the manuscript. MBV and MJS critically revised the manuscript. All authors read and approved the manuscript for publication.

## Supplementary Material

Additional file 1 **Search method**. A summary of search strategy and search terms for the identification of articles with MH. Click here for file
