# Supplementary material for: Benefits and risks of manual hyperinflation in intubated and mechanically ventilated intensive care unit patients: a systematic review
Source: Crit Care. 2012 Aug 3;16(4):R145. doi: 10.1186/cc11457 (PMC3580733; doi:10.1186/cc11457)
Supplement: Additional file 1 — Search method. A summary of search strategy and search terms for the identification of articles with MH. [file cc11457-S1.DOC]

Manuscript 1528250053639674 – revised version

Version date 200512

**Appendix to:**

**Benefits and Risks of Manual Hyperinflation in Intubated and Mechanically Ventilated Intensive Care Unit–patients – A Systematic Review**

Frederique Paulus^1^, Jan M. Binnekade^1^, Margreeth B. Vroom^1^ and Marcus J. Schultz^1,2^

**Academic Medical Center, University of Amsterdam, Amsterdam, The Netherlands:**

^1^Department of Intensive Care Medicine

^2^Laboratory for Experimental Intensive Care and Anesthesiology (L·E·I·C·A)

**Correspondence**

F. Paulus

Department of Intensive Care

C3–323

Academic Medical Center

University of Amsterdam

1105 AZ Amsterdam

The Netherlands

Phone: 31 205662509

Fax: 31 20 5669568

E–mail: [f.paulus@amc.uva.nl](mailto:f.paulus@amc.uva.nl)

**Search strategy**

The search strategy was first developed for Medline to be adapted later to search Embase and Cochrane/Dare. The following keywords (MeSH and text words) were used: “critical care”, “intensive care”, “manual hyperinflation”, “hyperinflation”, “bagging”, and “bag squeezing”. In addition, we used the keywords “hyper–oxygenation”, “physiotherapy”, and “physical therapy”. The initial search strategy was designed for maximal retrieval and there was no limitation on the type of study design to be identified.

**Search terms**

("intensive care"[MeSH Terms] OR ("intensive"[All Fields] AND "care"[All Fields]) OR "intensive care"[All Fields]) OR ("critical care"[MeSH Terms] OR ("critical"[All Fields] AND "care"[All Fields]) OR "critical care"[All Fields])

AND

((hyperinflation OR hyperinflation/hyperoxygenation OR hyperinflation/inflation OR hyperinflation breaths OR hyperinflation induced OR hyperinflation maneuvres OR hyperinflation methods OR hyperinflation parameters OR hyperinflation technique OR hyperinflation techniques) OR (manual hyperinflation OR manual hyperinflation maneuvres OR manual hyperinflations OR manual hyperventilation OR manual hyperinflation procedure))
